# Exploring the Content of Post-Traumatic Stress Symptoms among Parents after Paediatric Stem Cell Transplant

**DOI:** 10.1371/journal.pone.0126905

**Published:** 2015-05-12

**Authors:** Ulla Forinder, Lovisa Claesson, Katharina Szybek, Annika Lindahl Norberg

**Affiliations:** 1 Department of Social Work, Stockholm University, Stockholm, Sweden; 2 Department of Women's and Children's Health, Childhood Cancer Research Unit, Karolinska Institutet, Stockholm, Sweden; 3 U-CARE/Clinical Psychology in Heathcare, Department of Public Health and Caring Sciences, Uppsala University, Uppsala, Sweden; Central Institute of Mental Health, GERMANY

## Abstract

In the present study the aim was to explore the content in a trauma reported in a self-report questionnaire by parents of children with a life threatening illness. Semi-structured interviews were performed, with the aim to explore the specific cognitive and behavioral content of the trauma related symptoms reported by the individual informant. The transcripts of the interviews were analyzed with content analysis using a direct approach with a-priori categories according to the B and C categories of the DSM-IV diagnostic criteria for PTSD. The results give us the picture of a complex situation, where the self-report instrument PCL captured a spectrum of qualitatively different cognitions. The parents described traumatic thoughts and images relating not only to experiences in the past (i.e., truly post-traumatic), but also to current stressors and expected future events.

## Introduction

For parents, the diagnosis of a serious disease in their child is a psychological and existential challenge. Learning that your child has a life-threatening disease is recognized as a traumatic stressor with the potential to cause post-traumatic stress disorder (PTSD) [1]. In recent decades, we have seen an increasing body of studies examining the occurrence of PTSD and PTSD-like symptoms (PTSS) among parents of children who face serious threats to their general health [2], particularly in relation to a life-threatening condition such as cancer [3] in combination with an invasive medical treatment such as a stem cell transplant (SCT) [4]. SCT is a complicated treatment that has improved the prognosis of a number of fatal diseases. However, cure is still by no means ensured, and the procedure entails the risk of serious sequelae such as cognitive impairment, fatigue, gastro-intestinal problems, concern for appearance, pain, secondary cancer [5]. PTSS are reported by a significant minority of parents even after the medical treatment of the child is successfully completed and the child survived [4].

Few professionals would question the traumatic potential of your child suffering a life-threatening disease. The disclosure of the child’s diagnosis is generally seen as the primary critical event, and parental traumatic stress symptoms are indeed generally most prominent immediately after the diagnosis [6]. In addition, particularly stressful treatment procedures and serious medical complications may also have a potentially traumatic impact on parents [7], and watching the child in fear and distress has been shown to predict parental PTSS [8]. Consequently, the specific nature of the trauma is not immediately obvious because the situation involves a number of different types of severe stressors. In other words, it is not clear which specific aspects of the experience trigger the traumatic stress process.

Moreover, less severe stressors continue to disrupt parents’ lives even after the successful completion of the child’s treatment, e.g. issues related to the child’s health, school performance, and social life, as well as to the family finances. In research on parents of children with cancer, it has been suggested that parental self-reported distress captured with PTSS screening instruments may often also include responses to concurrent stressors [9]. It has been proposed that the general models for the psychological trauma process do not fully describe this situation [10]. Another characteristic that makes this type of trauma quite different from, for example, accidents, violence and natural disasters is that the medical care is present before and during, as well as after the traumatic event. This gives us a unique opportunity to intervene. As visualized in a model outlined by Kazak and colleagues [7], interventions for traumatic stress among patients and parents in the paediatric medical setting should not be restricted to the treatment of manifest PTS responses. We should also work preventatively by paying attention to the subjective experience of the traumatic event. To guide the development of such prevention, it would be valuable to improve our understanding of the nature of this particular type of trauma.

The present paper reports from a qualitative study with the overarching aim of improving our understanding of medical traumatic stress by exploring the content of post-traumatic stress symptoms in the form of intrusive cognitions and avoidant behaviours reported by parents of children who had undergone SCT.

## Methods

### Sample

The present results are based on data collected in a nationwide Swedish project that longitudinally investigated different aspects of the psychological consequences for parents whose children had undergone SCT and were alive at the time of the data collection [11–13]. Self-report questionnaires were completed on two occasions, 18 months apart.

The project included all parents in Sweden who met the inclusion criteria, which were: being the custodian of a child up to and including 18 years of age who had undergone SCT at least six months prior to the data collection, and sufficient knowledge of the Swedish language to understand the questions in a self-report questionnaire. For the first assessment, a total of 421 questionnaires were sent out, of which 284 completed questionnaires were returned (161 mothers and 123 fathers), thus, a response rate of 67%. There were 277 parents eligible for the second assessment (T2), having completed the T1 questionnaires, and whose children were alive. At T2 the response rate was 61% (n = 170; 99 mothers and 71 fathers). A more detailed description of the whole project are published earlier [13].

The overall project, including five studies, has a mixed method design, assessing the emotional wellbeing among the parents with both quantitative and qualitative methods [14]. However, the study we are presenting in this paper is purely qualitative, using interviews for data collection and qualitative content analysis in the analysis process. In the present study, parents were included if at T2 they reported symptoms indicating potential PTSD and agreed to participate in a telephone interview. Twenty six parents (15% of the participating parents) reported potential PTSD, and 17 of them agreed to be interviewed. Thus, 17 parents (10 mothers, 7 fathers) of 15 children were interviewed. The children’s medical conditions that had prompted SCT were leukaemia (n = 11), immunodeficiency (n = 2), solid tumour (n = 1), neurological disease (n = 1). Between 3 and 13 years had passed since SCT.

### Procedure

Invitations to participate in both T1 and T2 were sent by mail in a letter that included written information, a questionnaire, a slip that recipients could return if they chose not to participate, and a prepaid reply envelope. Reminders were sent by mail, two and four weeks after the invitations were sent, to those who had not returned the questionnaire or the slip. The questionnaire included a box that parents could check if they agreed to participate in a telephone interview, should we require more information. The study was approved by the Regional Ethical Review Board in Stockholm and performed in accordance with the ethical standards of the recent version of the 1964 Declaration of Helsinki.

### Assessment of potential PTSD

Data on PTS symptoms were collected using the PTSD Checklist Civilian Version (PCL-C) [15], assessing occurrence of symptoms of re-experience, intrusive cognitions, avoidance, numbing of responsiveness and arousal over the course of the past month. The sub-scales correspond to the three symptom clusters of PTSD according to the DSM-IV [1]: re-experience (5 items), avoidance (7 items) and hyper-arousal (5 items). Eight questions explicitly relating to the trauma (items 1–8) were phrased to specifically apply to the child’s illness and treatment.

To identify potential cases of PTSD, we used the PCL-C symptom criteria method [15], i.e. parents who answered ≥ 3 (moderately or more) for at least one symptom of re-experience, three symptoms of avoidance and two symptoms of hyperarousal. This method has shown diagnostic effectiveness for PTSD when compared to the Structured Clinical Interview for DSM-IV, PTSD module, among mothers of childhood cancer survivors [16].

### Interviews

Semi-structured interviews were performed by two psychologist students (Author 2, Author 3) under the supervision of two seniors: a registered psychotherapist (Author 1) and a registered psychologist (Author 4). Telephone interviews was chosen as an appropriate and efficient way to collect data, as we were not going to take non-verbal communication into consideration [17]. Sixteen parents were interviewed by telephone, and, furthermore, one via online chat, due to hearing difficulties.

The aim of the interviews was to explore the specific content of the context-related symptoms (PCL-C item 1–8) reported by the individual informant. Each interview involved questions about the symptoms which bothered the informant at least moderately, i.e. items which they had scored 3 or above. This interview approach resembles the think-aloud probing technique, based on cognitive theory [18]. The purpose of the method is to understand how respondents appraise questions, to capture the process behind the given answers and get a deeper knowledge of the content of the answers. As an example, a question could be “In the questionnaire you chose the answer ‘Quite a bit’ for the question ‘have you been avoiding activities or situations because they remind you of your child’s illness and treatment?’. Can you tell me what kind of activities or situations was in your mind when you answered that question?” Informants were thus encouraged to describe the content of distressing cognitions, dreams and reactions, and various ways of avoidance that came to their minds when answering the questionnaire, in line with the think-aloud method. Clarifying questions were used to deepen the answer. The duration of the interviews was between 30 and 100 minutes, and the interviews were recorded and transcribed verbatim by the interviewer.

### Analysis

The transcripts were analysed using directed qualitative content analysis [19]. A deductive approach was used, i.e. *à-priori* categories were formulated according to the B and C categories in the DSM-IV diagnostic criteria for PTSD [1] and the corresponding items 1–8 in PCL-C. This approach is appropriate when the structure of the analysis is operationalized on the basis of previous knowledge and is theory-testing [20]. Ten *à-priori* categories were formulated: Distressing **memories** of the child’s disease or SCT; Distressing **thoughts** (not including memories) relating to the child’s disease or SCT; Recurrent distressing **dreams** relating to the child’s disease or SCT; Perception of **re-experiencing** the child’s disease or SCT; Intense **psychological distress** at exposure to internal or external cues related to the child’s disease or SCT; **Physiological reactivity** on exposure to internal or external cues related to the child’s disease or SCT; **Cues** that induce reactions according to the B4 and B5 categories; **Avoidance** of thoughts, feelings or conversations related to the child’s disease or SCT; **Avoidance** of activities, places or people that induce recollections of the child’s disease or SCT; **Inability to recall** an important aspect of the child’s disease or SCT.

The analyses did not take additional verbal information into account, such as tone of voice; rather, they included only the manifest verbal content of the statements. Initially, the interviewers (Author 2, Author 3) read the interviews independently and coded all relevant statements according to the categories. Subsequently, the interviewers switched interviews and again coded them again, without knowing their colleague’s previous coding. Coding differences were thus revealed, and could be discussed and negotiated. Subcategories were created within each category, framed as different aspects of the main category. During this phase, the two senior researchers (Author 1, Author 4) supervised the analysis process. In a second phase, Author 1 and Author 4 conducted confirmatory analyses. This was done by reading, independently of one another, ten randomly selected transcriptions for each of the *à-priori* categories, and systematically investigating agreement with the previous analysis. A disagreement was found for a few subcategories, which were then re-coded. This analytical procedure could be referred to as a form of research triangulation, including the initial independent coding, and the complementary confirmatory analyses. It is reasonable to assume that this process increased the trustworthiness of the analysis [21].

## Results

The aim of this study was to gain a deeper knowledge of the content of the intrusive cognitions and avoidant behaviours reported by parents in relation to the illness and SCT treatment of their child. Ten *à-priori* main categories were used to guide the analyses. In total, 39 subcategories were found. In Table 1 we present the categories and the subcategories. In addition, Table 1 includes the total number of parents who were interviewed distributed by each main category, i.e. the parents that answered “moderately” or more on the item corresponding to the category in question. The running text includes quotations which illustrate the categories.

**Table 1:** Results from the analyses of the interviews: A-priori categories and subcategories.

Á-priori categories with reference to DSM-IV B and C symptom criteria for PTSD	No of inter-views*	Results; subcategories
B1. Distressing **memories** of the child’s disease or SCT.	16	Memories of medical events
		Memories of own distress
		Memories of realizing the seriousness of the disease
		Memories of siblings’ distress
B1. Distressing **thoughts** [not including memories] relating	16	Thoughts about recurrence of the disease
to the child’s disease or SCT.		Thoughts about siblings falling ill
		Thoughts about current problems
		Thoughts about the child’s future
		Thoughts about inadequate parenting
		Thoughts about loss
		Retrospective understanding of threat to the child’s life
B2. Recurrent distressing **dreams** relating to the child’s	7	Dreams in which the child is harmed or dies
disease or SCT.		Dreams of an actual stressful event
		Dreams of powerlessness
		Restless sleep
B3. Perception of **re-experiencing** the child’s disease or	10	Indications of possible recurrence of the disease
SCT.		Strong memories
B4. Intense **psychological distress** at exposure to internal	10	Fear
or external cues related to the child’s disease or SCT.		Depression
		Anger
		Shame and guilt
		Powerlessness
B5. **Physiological reactivity** on exposure to internal or	12	Restlessness
external cues related to the child’s disease or SCT.		Fatigue
		Crying
		Nausea
		Physiological stress reactions
B4 and B5. **Cues** that arouse reactions according to B4 or	15**	Cognitions
B5.		Conversations
		Disease, death and medical care
		Previously neutral stimuli
		The child
		Documentation from the period of disease or SCT
C1. **Avoidance** of thoughts, feelings, or conversations related to the child’s disease or SCT.	10	Avoiding distressing thoughts and feelings by keeping oneself occupied
		Avoiding social interaction
C2. **Avoidance** of activities, places, or people that arouse	5	A wish to avoid (medical check-ups)
recollections of the child’s disease or SCT.		Physical avoidance
C3. **Inability to recall** an important aspect of the child’s.	13	Deliberately avoiding certain distressing memories
disease or SCT		Difficulties to discern details from the treatment period

* Total number of parents that did answer “moderately” or more on the item corresponding to each á-priori category.

** Parents that did answer “moderately” or more on at least one of the items that concern the criteria B4 and B5.

### Memories

This category includes narratives of memories of incidents that took place during the child’s illness and treatment and which had caused distress to the parents over the course of the past month. The character of the memories differed. Some described fragments or images which passed quickly through the mind; others described them as like films playing out. Some parents mentioned that the memories were always there and were difficult to get rid of. A few described how they became absent-minded when the memories occurred.

The majority of the parents reported unpleasant memories relating to medical events which involved their child suffering. The content of these memories related to acute and unwanted events, e.g. the onset of illness, severe allergic reactions, emergency situations, horrid symptoms and erroneous treatment, but also planned parts of the treatment such as radiation, chemotherapy and isolation. What the situations appearing in the memories had in common was that they were appraised by the parents as threats to their child. “They made facemasks because the radiation was directed right at his head. He had to lie completely still and then they gave him the radiation over the course of a week. To lie there and take that, something that is actually deadly.”

Most of the parents also reported memories of their own reactions during the child’s illness and treatment. These memories were painful as they once again induced feelings of powerlessness, fear, loneliness, uncertainty and other strong negative reactions. “I was so bloody afraid, and Kate was so unwell. I was so bloody sad, alone, the incredible powerlessness—thinking what the hell are we going to do?”

Furthermore, the moment of realization that the diagnosis has serious implications was reported as a distressing memory by the majority. For some, this moment of realization came immediately following the disclosure of the diagnosis, while for others it came when they were exposed to other ill children or when the prognosis was communicated during treatment.

Finally, memories of siblings in distress were described, in particular those memories concerning their exposure to the ill sibling’s suffering and parental neglect. It is worth noting that the awareness that siblings had been suffering generally did not seem to surface fully in parents’ minds until the medical situation had stabilized.

### Thoughts

This category includes distressing thoughts relating to the child’s illness or treatment. Although the item in the questionnaire explicitly asked for thoughts about an earlier event, several parents described that they perceived thoughts concerning the present and the future to be relevant to this item.

The thoughts were often accompanied by feelings of fear and uncertainty. The awareness that the disease may actually recur was common among the parents and induced worry that became worse at the medical check-ups. For a few parents, the distressing thoughts associated with the child’s illness and treatment also involved worry about the health of siblings. Many parents also reported distressing thoughts about the child’s current problems and suffering in relation to, for example, sequelae and poor school performance. Parents expressed that there was still suffering and there were still threats to the child’s health and wellbeing. “The danger has not passed as he has had complications.”

Thoughts about the child’s current problems in some way related to their future prospects. In addition, explicit thoughts about the child’s future were playing on the minds on some of the parents. These thoughts expressed worry about the child’s social life, studies, family situation and job prospects. “It’ll be harder when they get older, in two weeks she’ll be 19 and it’ll become harder, with friends and what not. You wonder, ‘how can she do well, how can she get on in life?’”

Several parents described thoughts about inadequate parenting, including existential ideations of not being able to protect their children from pain and fear. This related to the painful and distressing events the ill child had endured, but also the distress of siblings. As mentioned above in the category Memories, parents generally did not seem to have been fully aware of the distress siblings had suffered until the situation for the ill child had stabilized, and this delay seemed, in itself, to induce feelings of guilt. In other words, the memories of siblings’ distress generated thoughts about being an inadequate parent and subsequent feelings of guilt. “It’s really hard for me to deal with and what is most hard for me is that I didn’t stand up and protest. That I allowed anyone else to terrify Tom.”

Thoughts about loss were described by some parents. This related to the child’s loss of a normal childhood and the parent’s loss of a normal parenthood and of the child they once had. “I was robbed of a happy time with my baby.” In addition, some parents described a feeling that they had lost their previous personality or identity. Many parents mentioned that the experience changed their personality—for the worse. They missed their “old self” and some recognized this had happened to their partner. “My life changed. I became a completely different person. So I understand why the divorce rate is high among parents who have been through this. So may sides of you come to the surface that you didn’t know you had.” The thoughts about loss sometimes included ruminations on the injustice that the illness and sorrows were to them.

Retrospective understanding of the threat to the child’s life was pointed to by a few parents. They were now thinking back on their experiences and realizing the life-threatening danger their child had been exposed to. Parents described the experience of this threat vividly, almost as if the threat was in the present and not something in the past. “It catches up with you, that he could actually have died and that it was a serious as it was.”

### Dreams

About half of the parents reported having experienced distressing dreams associated with the child’s illness and treatment over the course of the past month. Such dreams were often described as rich in detail and distinct, and, in that sense, somewhat different from other dreams. It was also mentioned that the complete course of the dream was experienced before awakening. A few parents reported waking up screaming or with physical arousal manifesting as cold sweat and heart palpitations. Some dreams included fictional threatening situations where either the child or siblings died or were hurt in accidents or because of violence. Other dreams were detailed memories of actual events from the time when the child was ill or undergoing treatment. Feelings of powerlessness were often connected to distressing dreams, and parents associated this with the powerlessness they had felt during the child’s illness and treatment. In addition, one parent reported restless sleep without discernible dreams in this category.

### Re-experiencing the child’s disease or SCT

Several parents reported being troubled by re-experiencing. However, it is questionable whether any of the reports actually reflected flashbacks in the true sense; rather, the interviews revealed that reports of re-experiencing appeared to reflect intense reactions triggered by a suspicion that the disease had recurred. “… if she were to come to me and say that she has a symptom… my body will get going without me having the time to think.” In addition, the reports of re-experiencing included descriptions of strong reactions to distressing memories, which often occurred suddenly. “You feel that you can almost sense the smell, what would otherwise vanish and be dismissed, you see it really clearly.”

### Psychological distress

According to the category concerning intense psychological distress at exposure to cues related to the child’s disease or SCT, five subcategories representing different aspects of emotion were identified: fear, depression, anger, shame and guilt, and powerlessness. The emotional reactions were occasionally perceived as uncontrollable. A few parents used words signifying pain, such as “grating”, “painful”, “suffering” and “draining”. Being tired or strained for other reasons intensified the reactions.

Most of the parents mentioned fear, sometimes in the form of agony or a perception of threat. Depression triggered by the child’s illness and treatment was also reported by a majority of the parents. Words indicating grief, disappointment and a feeling of emptiness or heaviness were used. “There is a lot of grief, it is hard, it is really hard for me to get through the day.” Metaphors that expressed a feeling of falling or being pulled down were also placed in this category. Depressive reactions were often induced in connection with thoughts about loss or suffering that the child or the parents had experienced previously. “This thing with the diagnosis, I fell apart completely. And the memory of that, when I think about it I become extremely sad and cry.”

Anger, sometimes expressed as irritation or frustration, was reported by many of the parents. Perceptions of injustice seemed to be the most common trigger for anger. “I still have a sort of anger towards other mothers who have healthy children.” Moreover, some parents mentioned shame and guilt, including feelings of humiliation and remorse, which primarily came about through self-criticism or thoughts about the disapproval of others. “It sets off strong reactions that I can’t stop. And I can think that it feels humiliating, I feel small and feeble—it’s hard feeling like this and I don’t like it at all.” Finally, a few parents reported powerlessness, a sense of not being able to protect their child from harm. This sub-category also covered feelings of vulnerability and weakness.

### Physiological reactivity

Most of the parents reported physiological reactivity on exposure to internal or external cues related to the child’s disease or SCT. For some, the physiological reactions were temporary, for others they were more long-lasting. “You feel bad constantly, pressure on your chest, trouble breathing, but they don’t find anything wrong with me, it’s like I’m standing in a *cul-de-sac* where there’s no medical cause for my problems.”

The analysis yielded five subcategories of physiological reactivity: restlessness, fatigue, crying, nausea and physiological stress reactions. The majority of the parents reported having experienced the latter, e.g. palpitations, sweating, tension, loss of appetite and chest tightness. “And so you think ‘Shit! Why is my heart racing now? Why do I feel so warm now?’ And then you realise that you’re thinking about his illness.”

### Cues triggering psychological and physical reactions

Cues that were described as triggering reactions in accordance with the symptom criteria B4 or B5 were organized into a separate category, defined as internal or external cues that symbolized or resembled events experienced over the course of the period the child was ill or undergoing SCT, and that provoked intense psychological distress or physiological reactivity. The cues mentioned by the parents were classified into six subcategories: cognitions; conversations; disease, death and medical care; previously neutral stimuli; the child; and documentation from the period of disease and SCT.

Cognitions, i.e. thoughts, memories or dreams about future recurrence of the child’s disease or the risk that the child would die were mentioned by the majority of the parents as triggering fear and physical stress reactions. “That he will get sick again and won’t be able to get through it …. If that were to happen again, there’s maybe only one way out, because as far as I can see there’s not much more that medicine can offer. Of course, it’s a huge fear: what happens to you, to the siblings? Will we cope?”

Several parents mentioned that they became distressed by conversations that touched on certain issues, especially conversations about memories from the period the child was ill or undergoing SCT, or questions about the child’s current health condition. “We live in a small community and people often ask questions.”

The majority of the parents said that they overreact to stimuli related to disease, death and medical care in general, but, in particular, reactions were triggered by events and routines associated with the child’s health. “… he takes a lot of medicine every day and what not, and you get to thinking.” The child’s routine check-ups at the hospital caused distress. “… we are at the hospital a lot, so we are confronted with that environment several times a year. That’s why I can’t put any distance between us and that.” However, cues involving disease and death happening to other people were also potential triggers, for example, stories about disease or death in the media, a logo for a childhood cancer support organization or illness in the families of friends and colleagues. “It may be that someone calls into work to say that they can’t come in because their child is ill.”

Furthermore, parents mentioned that a previously neutral stimulus that had been connected to a traumatic event could become a cue for distress. “We avoid listening to this music because it immediately reminds us of those times, of hard times.”

Features of the child itself were described as triggers of distress. Specifically, indications of possible recurrence of the disease induce fear and worry. In addition, parents described that the after-effects of the disease or treatment on the child’s appearance, behaviour or health not only triggered painful memories, but also worries and concerns directly related to these after-effects. “When you have one of these bone marrow transplants, you become sterile. When she hear this—she was really sad.” The impact of the disease and the SCT were specific, as well as general. “So, of course, there are also complications after this sort of thing. There has been an impact on his demeanour. We’re having to get to know a different person.” A divorced parent who had shared custody reported that the child’s recurrent absence triggered negative emotional reactions.

Finally, parents mentioned that documentation such as photos and videos from the period of illness and treatment brought up stressful memories and reactions.

### Avoidance of thoughts, feelings or conversations related to the child’s disease or SCT

Avoidance of cognitions and emotions is, by definition, a bit problematic to determine through self-reports. However, intentions and strategies used to avoid thoughts and feelings were reported by a majority of the parents in the interviews.

Keeping yourself occupied was one way of cognitive and emotional avoidance. “In principle, I’m doing something constantly. It’s rare that I’m still, I busy myself with something that takes concentration.”

In addition, several parents reported avoiding conversations about medical issues in social situations, for example, at work, to avoid questions about the child’s condition. This avoidance of conversations did sometimes reduce their willingness to seek professional psychological help.

However, avoidance of conversations about the child seemed to have various starting points. Parents stated that other people do not understand and so they get weary of trying to explain, and that the stories make people scared. “You feel that people don’t understand, I walk away from it. I’ve become very antisocial as a result of this experience because of my memories of this.” Parents also described avoiding conversations because they wanted to leave the disease and SCT behind and move on. “I have talked a lot about this and it feels like it needs to stop.”

### Avoidance of activities, places or people that induce recollections of the child’s disease or SCT

In the category relating to physical avoidance, both actual and desired avoidance were included. The situation that was by far the most frequently mentioned was medical check-ups, which several parents expressed that they wish they could avoid. One strategy was to let the other parent accompany the child to the check-up. However, in the end, few of the parents neglected to go to the check-ups. “There have been a number of occasions that we have called up and said, ‘no, we can’t come because he’s ill’. Then, when you do go, we usually take our car so we can get away from these as quickly as possible.”

Moreover, one parent mentioned that the family were no longer listening to certain music or visiting places that they had enjoyed before and during the child’s treatment. “We avoid certain places that we went to before she got ill, we have wonderful memories of these places, but we avoid going to them.” Parents also mentioned that they sometimes avoided their children’s involvement in certain activities because they felt unable to manage the worry it caused them. “I don’t want him to get into situations where something can happen.” One parent described avoiding getting close to the child, feeling distressed to see her suffer. “Every time she is going to have a bone marrow test, there’s uproar, she is so afraid that there will be a relapse. So I try to avoid being in there with her, I go out into the garden because I don’t want to confront her. I can’t do it, I feel awful when I see her suffering that much.”

In addition, certain answers to the question about avoiding activities or places as a consequence of the child’s disease and treatment turned out to describe a loss of interest in leisure pursuits, social activities or taking care of the home. These narratives reflected melancholy or listlessness, rather than avoidance.

### Inability to recall an important aspect of the child’s disease or SCT

This category included statements that indicate an inability to recall a traumatic incident from the period of the child’s treatment and illness. Parents reflected that this ability to not remember the most difficult situations may serve as a defence that protected them.

However, only a few of the answers to this question seemed to reflect true post-traumatic amnesia, and these related to the moments of shock upon first being informed of the diagnosis. “They said, ‘you have to come into hospital, it’s leukaemia’, I don’t remember much after that.”

Most of the answers to this question were found to reflect either a deliberate avoidance of distressful memories or an inability to remember less significant events or the course of events that happened a long time ago. “Different parts of the treatment period, medicines, bone marrow, his allergic reactions, blood poisoning… There is so much I can’t remember.”

## Discussion

The aim of the present study was to explore the contents of post-traumatic stress symptoms reported by the parents of children who had undergone SCT. The results give us a picture of a complex situation in which the self-report instrument PCL captured a spectrum of qualitatively different cognitions. The parents described traumatic thoughts and images relating not only to experiences in the past (i.e. truly post-traumatic), but also to current stressors and feared future events. We consider the acute phase of disease and SCT passed for all the parents in the present sample, for which between 3 and 13 years had passed since SCT. Accordingly, traumatic thoughts and images relating to current stressors seemed primarily to involve more or less permanent late effects in the child. Moreover, traumatic cognitions included both concrete events they had previously experienced, or expected to happen, and existentially-coloured insights into the transience of life. In addition, we think it is worth noting that flashbacks were rare, if there were any at all. Indeed, a number of parents reported reactions they related to as flashbacks; however, the analyses revealed that the word flashback in everyday language seems to denote strong emotional and bodily reactions evoked by painful memories, rather than flashbacks in the psychological sense. We also find it important to highlight that the child’s pain and discomfort were important sources of suffering for the parents. A previous study has demonstrated that pain is a long lasting effect among children after SCT [22]. Pain in children is, to our knowledge, something that clinicians are well aware of, and our findings confirm the importance to acknowledge pain.

Furthermore, one particular type of traumatic memory that parents mentioned was the disclosure of the child’s diagnosis. It should be underscored that the delivery of bad news is a crucial communication event with the potential of evoking severe distress in patients and their loved ones. Supervision by a psychosocial professional can be of great benefit for health care providers meeting this situation.

Parents reported the somewhat exaggerated and dysfunctional types of cognitions usually seen in association with PTSD, e.g. apprehension that something terrible might happen to the child or excessive fear that another family member would fall ill. At the same time, certain aspects of their concerns reflected a real and imminent threat. Worry about the health of siblings may be reasonable as many of the illnesses for which SCT is performed have genetic components. Relapse of the disease and negative long-term sequelae are also real threats. We have previously shown that the severe late effects that follow SCT in childhood may lead to psychosocial problems and a poorer quality of life as an adult [23]. McCarthy and colleagues [6] found that the child’s current quality of life was associated with their parents’ self-reported PTS symptoms. It is reasonable that parents’ realistic worries about the present or the future may generate high scores in a cancer-related PTS self-report instrument, although the intention is to assess reactions to events in the past. Thus, we may not be able to determine whether the reported symptoms reflect current or post-traumatic stress. In addition, currently ongoing stress is likely to complicate the process of coping with post-traumatic stress [10]. In their cognitive model of posttraumatic stress disorder, Ehlers and Clark [24] suggest that PTSD is maintained by a sense of current threat. The influence and consequences of actual current threat on the PTS process have yet to be sufficiently investigated, since it should impact the general approach to PTSD monitoring. Particularly, this should be recognized in relation to severe and potentially fatal disease.

Another type of cognition that induced distress had an existential quality. Parents described how the earlier experiences had often been reinterpreted and appraised differently over time, leading to, for example, an understanding of the full extent of the threat posed by their child’s illness, or a realization of how their other children had been suffering. Rather than being specific memories, these appear to have been insights that had emerged gradually, typically surfacing after the child had recovered. This lengthy process brings to mind the social science concepts *biographical disruption* and *narrative reconstruction*, which describe the impact of serious disease on the individual as a process that takes place over time [25]. It may also be compared with the notion that PTSD sometimes seems not to arise until the person feels that the imminent threat has decreased. Moreover, there seems to be a mutual influence between negative cognitive appraisals and PTS symptoms [24,26].

The present study has some advantages in that it is nationwide and includes fathers as well as mothers. However, there are certain methodological issues to be discussed. We have focused on parents’ experiences in connection with specific PTSD symptoms and we did not consider experiences outside this focus. We regarded this approach as the most appropriate for our aim, although a more explorative approach might have discovered additional experiences. Moreover, in the analysis, we only used the manifest material as the parents presented it. Thus, we had no intention to interpret any additional verbal material as sighs and tone of voice, nor did we use visual information such as facial expressions and body postures. Some issues may be easier to discuss on the phone than in a face-to-face situation. In addition, telephone interviews have, when used with structured self-reports, proved to give results equal to those of pen-and-paper [27]. For these reasons, we considered telephone interviews convenient and appropriate. Furthermore, eight out of the twenty-six parents who reported symptoms indicating potential PTSD chose not to participate in the study. We will never know whether these eight parents had alternative experiences to those who took part in the interviews. As in the case of qualitative studies the question about generalisations is a question of transferability [28]. We believe that the findings in this study could be transferred to other groups of parents.

In summary, the present findings further highlight the complexity of the long-term process of stress, strain and trauma in medical traumatic stress. It should be acknowledged that reactions to current stressors are qualitatively different from post-traumatic stress, and the two are likely to require different therapeutic approaches. In the continued effort to understand medical traumatic stress, it would be useful to develop a context-specific prevention and treatment model targeting the different aspects of the situation. Until such a treatment model is available, existing interventions should be offered to seriously ill patients and their loved ones, not only in the acute crisis, but as part of the long-term follow-up. It is a striking fact that several parents still suffer from untreated severe stress years after their child has undergone SCT and survived.

## Conclusion

The results give us the picture of a complex situation, revealing different cognitions behind the self-report responses to the instrument PCL. The parents described traumatic thoughts and images relating not only to experiences in the past (i.e. truly post-traumatic), but also to current stressors and expected future events. This may indicate a risk of overestimation of the PTSD rates in this population when using checklists alone, since the suffering may as well be a consequence of present stressors.
